# Adenosine-A_3_ receptors in neutrophil microdomains promote the formation of bacteria-tethering cytonemes

**DOI:** 10.1038/embor.2013.89

**Published:** 2013-07-02

**Authors:** Ross Corriden, Tim Self, Kathryn Akong-Moore, Victor Nizet, Barrie Kellam, Stephen J Briddon, Stephen J Hill

**Affiliations:** 1 Institute of Cell Signalling, School of Biomedical Sciences, Medical School, University of Nottingham, Nottingham NG7 2UH, UK; 2 Department of Pediatrics, Nottingham NG7 2UH, UK; 3 Skaggs School of Pharmacy and Pharmaceutical Sciences, University of California, San Diego, La Jolla, California 92093-0687, USA; 4 Centre for Biomolecular Sciences, School of Pharmacy, University of Nottingham, Nottingham NG7 2UH, UK

**Keywords:** neutrophils, adenosine receptors, host–pathogen interactions

## Abstract

The A_3_-adenosine receptor (A_3_AR) has recently emerged as a key regulator of neutrophil behaviour. Using a fluorescent A_3_AR ligand, we show that A_3_ARs aggregate in highly polarized immunomodulatory microdomains on human neutrophil membranes. In addition to regulating chemotaxis, A_3_ARs promote the formation of filipodia-like projections (cytonemes) that can extend up to 100 μm to tether and ‘reel in’ pathogens. Exposure to bacteria or an A_3_AR agonist stimulates the formation of these projections and bacterial phagocytosis, whereas an A_3_AR-selective antagonist inhibits cytoneme formation. Our results shed new light on the behaviour of neutrophils and identify the A_3_AR as a potential target for modulating their function.

## Introduction

Extracellular nucleotides/nucleosides are critical mediators of immune cell function [1]. Extracellular ATP has been proposed to act as a long-range ‘find me/eat me’ signal that promotes phagocytic clearance by leukocytes [2]. Indeed, extracellular ATP serves as an important autocrine/paracrine mediator of chemotaxis in several cell types [3, 4]. In particular, polarized release of ATP in response to chemoattractant stimulation has a critical role in neutrophil chemotaxis [3]. Although several studies have concluded that ATP itself does not act as a neutrophil chemoattractant [3, 5], the removal of endogenously released ATP by the enzymatic scavenger apyrase nevertheless inhibits the migration of these cells in response to chemoattractants, such as fMet-Leu-Phe (fMLP) [3]. Although the numerous potential fates of extracellular ATP [6] have made it difficult to pinpoint its precise role in the control of chemotaxis, inhibition of fMLP-mediated chemotaxis by addition of exogenous adenosine deaminase suggests that G-protein-coupled adenosine receptors are involved in the process [3].

On release, ATP is rapidly converted to adenosine by ecto-nucleotidases on the neutrophil cell surface [7], leading to activation of G-protein-coupled adenosine receptors. One member of this receptor family, the A_3_-adenosine receptor (A_3_AR), stands out as a key regulator of neutrophil function. A_3_AR-knockout mice show elevated bacterial counts in the peritoneum and peripheral blood after caecal ligation puncture [8]. In addition, neutrophils isolated from A_3_AR-knockout mice show impaired migration in response to chemoattractant stimulation [3]; indeed, a growing body of evidence suggests that A_3_ARs regulate processes that involve cytoskeletal remodelling (for example, chemotaxis) [3, 9]. Initial investigations into the cellular localization of neutrophil A_3_ARs suggested that they are distributed in a highly polarized fashion on the cell membrane [3]. However, until recently, the only widely available methods for investigating the localization of receptors (such as the A_3_AR) during typical neutrophil behaviours were cell fixation and immunocytochemistry or the overexpression of tagged receptors in model cell lines (for example, differentiated HL60 cells). Both techniques have severe limitations; the former does not permit imaging of living cells, while the latter is a relatively artificial model of primary human neutrophils.

Here, we have further investigated the localization and role of endogenous A_3_ARs in living human neutrophils using the recently developed fluorescent A_3_AR ligand CA200645 [10]. Using live-cell imaging, we found that endogenous A_3_ARs aggregate into plaque-like microdomains on human neutrophil membranes. In addition to facilitating cell migration, A_3_AR plaques were associated with membrane projections that could extend many times the lengths of the cells. Similar structures, termed ‘tethers’, ‘tubovascular extensions’ or ‘cytonemes’, can be observed using scanning electron microscopy and have been implicated in neutrophil adherence, migration, interactions with bacteria and the phagocytosis of labelled beads [11–14]. Here, we demonstrate the involvement of A_3_ARs in the formation of these structures, which enable neutrophils to sample, capture and ‘reel in’ pathogens for phagocytosis.

## Results and discussion

### A_3_ARs aggregate in plaque-like microdomains

**Figure 1:**
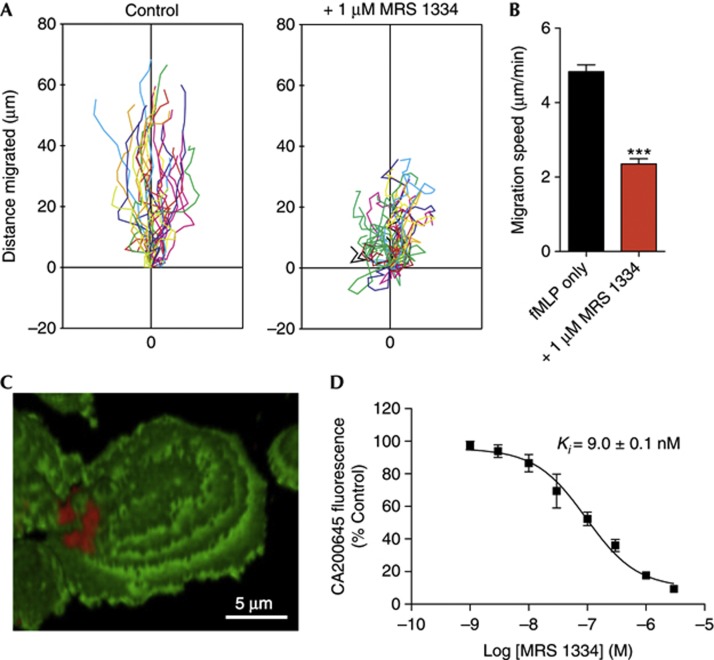
Endogenous A_3_ARs aggregate into plaque-like microdomains that regulate processes involving cytoskeletal remodelling. (**A**) Representative plots showing directional migration of neutrophils within fMLP gradients is inhibited by A_3_AR-selective antagonist MRS1334. (**B**) Quantification showing 51% reduction (***P<0.0001; Student’s *t*-test) of migration speed of MRS1334-treated cells (*n*=97–127 cells, three separate experiments). (**C**,**D**) Flow cytometry-based displacement experiments using CA200645 and unlabelled MRS1334 showed that the fluorescent ligand predominantly detected A_3_ARs. Data shown are means±s.e.m. of three separate experiments. A_3_AR, A_3_-adenosine receptor; fMLP, fMet-Leu-Phe.

In keeping with previous findings regarding the role of A_3_ARs in neutrophil migration, the highly selective A_3_AR antagonist MRS1334 [15], at a concentration of 1 μM, inhibited the migration of neutrophils in response to fMLP in a microscope-based migration assay (Fig 1A), reducing the average migration speed by approximately half (Fig 1B). We investigated the localization and role of endogenous A_3_ARs in living human neutrophils using the recently developed fluorescent A_3_AR ligand CA200645, a xanthine amine congener derivative connected via a linker to a BODIPY 630/650 fluorophore [10]. Confocal imaging of neutrophils incubated with CA200645 and the general membrane stain Vybrant DiO revealed that A_3_ARs aggregate into highly polarized, plaque-like microdomains (Fig 1C). As this polarized distribution made it difficult to assess the specificity of CA200645 binding by microscopy, we used flow cytometry to assess the total binding of CA200645 and investigate the pharmacology of these microdomains in live cells. Competitive binding experiments revealed that MRS1334 blocked the binding of CA200645 with a *K*_*i*_ value of 9.0±0.1 nM (*n*=4; Fig 1D). Given the ∼100,000-fold selectivity of MRS1334 for the A_3_AR compared with other adenosine receptor subtypes and the similarity of this value to the published *K*_*i*_ of MRS1334 at the human A_3_AR (2.7 nM) [16], we concluded that CA200645 predominantly detects A_3_ARs on human neutrophils.

### A_3_ARs promote the formation of membrane protrusions

**Figure 2:**
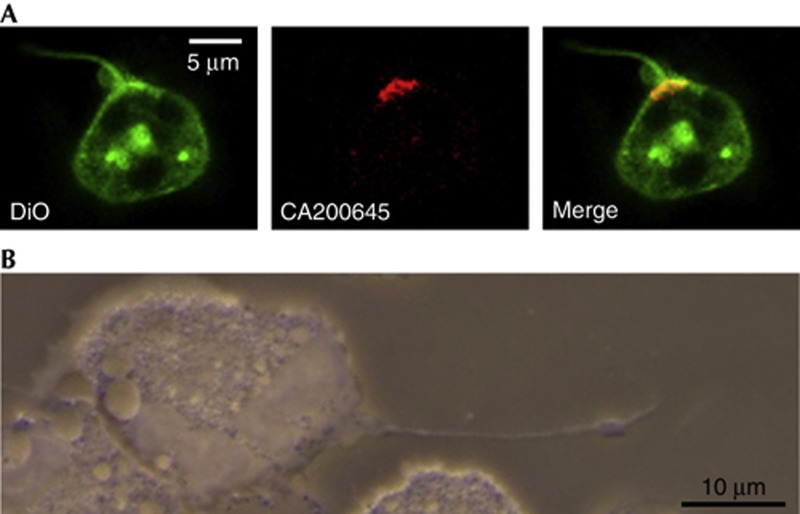
A_3_AR activation promotes the extension of membrane projections from human neutrophils. (**A**) Confocal imaging of Vybrant DiO/CA200645-labelled human neutrophils revealed that A_3_AR plaques were frequently associated with membrane projections. Quantitative analysis of neutrophils isolated from the blood of three different healthy volunteers revealed that 69.3±6.7% of these projections were associated with A_3_AR plaques. (**B**) A Riveal Contrast Microscope (Quorum, Ontario, Canada) was used to show that these projections are also found on unstained, phorbol 12-myristate 13-acetate-treated human neutrophils. A_3_AR, A_3_-adenosine receptor.

**Figure 3:**
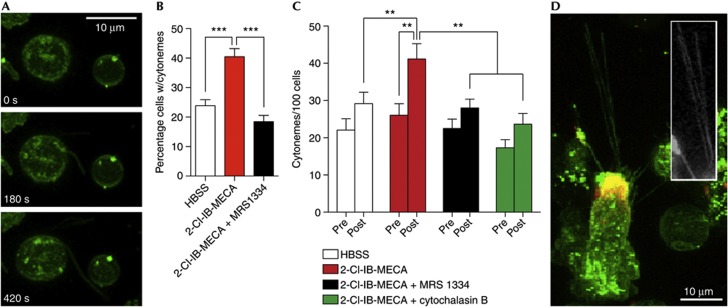
The A_3_AR-selective antagonist MRS1334 and cytochalasin B inhibit A_3_AR agonist-stimulated cytoneme formation. (**A**) The A_3_AR-selective agonist 2-Cl-IB-MECA (1 μM at *t*=0 s) promotes the extension of cytonemes from neutrophils. Quantitative analysis revealed that 2-Cl-IB-MECA treatment significantly increases the percentage of cells exhibiting cytonemes (**B**) and the number of cytonemes/cell (**C**), effects inhibited by MRS1334 or cytochalasin B. Data shown are means±s.e.m. of 26–35 individual experiments; significance was determined via one-way analysis of variance with *post-hoc* Newman–Keuls tests. (**D**) ATRA-differentiated HL60 cells, which exhibited both A_3_AR plaques and plaque-associated cytonemes (merged image of a Vybrant DiO and CA200645-labelled HL60 cell), were transfected with the fluorescent F-actin probe LifeAct, confirming the presence of F-actin in these structures (inlay, white). **P<0.01, ***P<0.001. A_3_AR, A_3_-adenosine receptor; HBSS, Hank’s Buffered Salt Solution; 2-Cl-IB-MECA, 2-chloro-N^6^-(3-iodobenzyl)adenosine-5′-N-methylcarboxamide.

Neutrophil A_3_AR plaques were often associated with long, thin membrane protrusions that were typically <500 nm in diameter and frequently exceeded 10 μm in length (Fig 2A). Analysis of images collected using neutrophils isolated from three healthy volunteers revealed that nearly 70% (69.3±6.7%) of these protrusions were associated with A_3_AR plaques. Structures with similar characteristics in neutrophils have been given a number of designations, including ‘tethers’ and ‘cytonemes’ [11, 13]. To determine whether A_3_ARs were involved in the formation of these structures, we used the potent, A_3_AR-selective agonist 2-chloro-N^6^-(3-iodobenzyl)adenosine-5′-N-methylcarboxamide (2-Cl-IB-MECA), which exhibits 1,400-fold selectivity for A_3_ over A_2a_ adenosine receptors [15]. We found that treatment with 2-Cl-IB-MECA (1 μM) promoted the rapid extension of these structures (Fig 3A). To quantify the tether-promoting effects of A_3_AR activation, we performed a series of experiments in which we treated neutrophils with either Hank’s Buffered Salt Solution (HBSS) or 2-Cl-IB-MECA, in the presence or absence of MRS1334 (1 μM) or the actin polymerization inhibitor cytochalasin B (300 nM). The data from these experiments were randomized, and the number of cytonemes per cell before and 7.5 min following HBSS or 2-Cl-IB-MECA treatment was determined by a blind observer using predefined criteria (described in the Methods section). The results of 34–35 individual experiments using neutrophils isolated from the blood of nine healthy volunteers (559–674 cells total) revealed that 2-Cl-IB-MECA promoted an increase in the percentage of cells exhibiting cytonemes (Fig 3B) and the number of cytonemes/cell (3C), effects that were blocked by MRS1334 (Fig 3D). Cytochalasin B also inhibited 2-Cl-IB-MECA-induced cytoneme formation (Fig 3C). Imaging of differentiated HL60 cells (which mimic neutrophil behaviour and show both plaques and cytoneme projections) expressing the filamentous-actin probe LifeAct revealed that these structures were composed of F-actin (Fig 3D). Neither MRS1334 nor cytochalasin B significantly affected cytoneme formation, as can be seen from the pre-agonist treatment bars in Fig 3C. Imaging experiments performed in the presence of the DNA stain SYTO 83 revealed that cytonemes were not associated with DNA-based neutrophil extracellular traps (supplementary Fig S1 online). Thus, neutrophil cytonemes are actin-based structures that protrude from A_3_AR-rich plaques whose formation can be stimulated in an A_3_AR-dependent manner.

### Cytonemes enable neutrophils to capture pathogens

**Figure 4:**
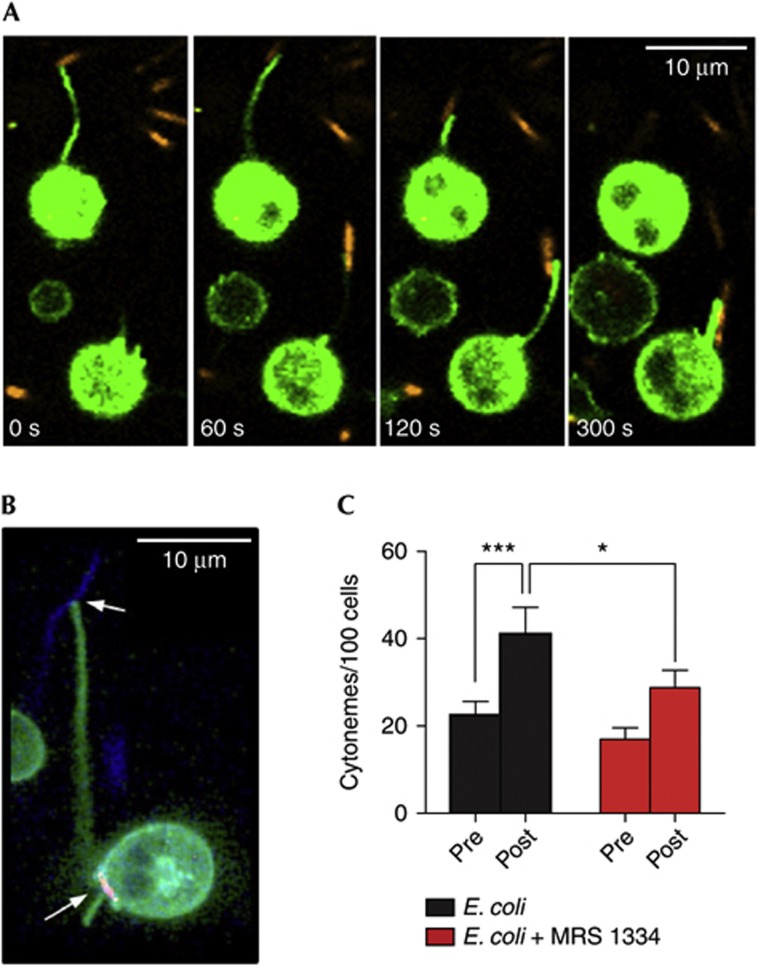
Neutrophil cytonemes formed in response to E. coli exposure are capable of tethering and ‘reeling in’ bacteria for phagocytosis. (**A**) Time-lapse confocal imaging shows neutrophils (Vybrant DiO labelled; green) can capture E. coli (SYTO 83 labelled; orange) via extension of cytonemes, which are rapidly retracted for phagocytosis. (**B**) Imaging of CA200645-labelled neutrophils showed bacteria-tethering cytonemes are associated with A_3_AR plaques (red); SYTO 83-labelled E. coli are pseudocoloured blue. (**C**) Exposure of neutrophils to E. coli promotes cytoneme extension, an effect inhibited by MRS1334 (*n*=26 individual experiments). Data shown are means±s.e.m.; statistical significance was determined via repeated measures analysis of variance with *post-hoc* Newman–Keuls tests. *P<0.05, ***P<0.001. See also supplementary Videos S1, S2 online. A_3_AR, A3-adenosine receptor.

Filipodia and nanometer-thin micropodial extensions (NMEs) have been shown to enable the capture and ‘reeling in’ of beads and pathogens in macrophages [17] and epithelial cells [18], respectively. In the case of NMEs, the capture of bacteria has a critical role in epithelial invasion and is driven by extracellular ATP [18]. Although cytonemes differ from NMEs (particularly in terms of maximum length), previous studies using scanning electron microscopy indicated that they might have a similar role in neutrophils [12]. We postulated that A_3_AR-induced cytonemes could represent a mechanism for the targeted, long-range capture or sampling of microbial pathogens. To test this hypothesis, we exposed human neutrophils to SYTO 83-stained *Escherichia coli*. Although some cells exhibited typical chemotactic or phagocytic behaviours, a number of cells rapidly extended cytonemes in response to bacterial exposure (supplementary Videos S1 and S2 online; green, neutrophils; orange, *E. coli*). The images shown in Fig 4A depict two cells rapidly extending cytonemes, capturing SYTO 83-stained *E. coli* and retracting the captured bacteria back to the cell surface. CA200645 labelling showed that these bacteria-tethering structures were associated with the A_3_AR plaques (Fig 4B). Quantitative experiments revealed that *E. coli* exposure approximately doubled cytoneme number (Fig 4C). This increase was suppressed by MRS1334, indicating a role for the A_3_AR in bacteria-mediated cytoneme extension.

### Cytonemes enable long-range capture of pathogens

**Figure 5:**
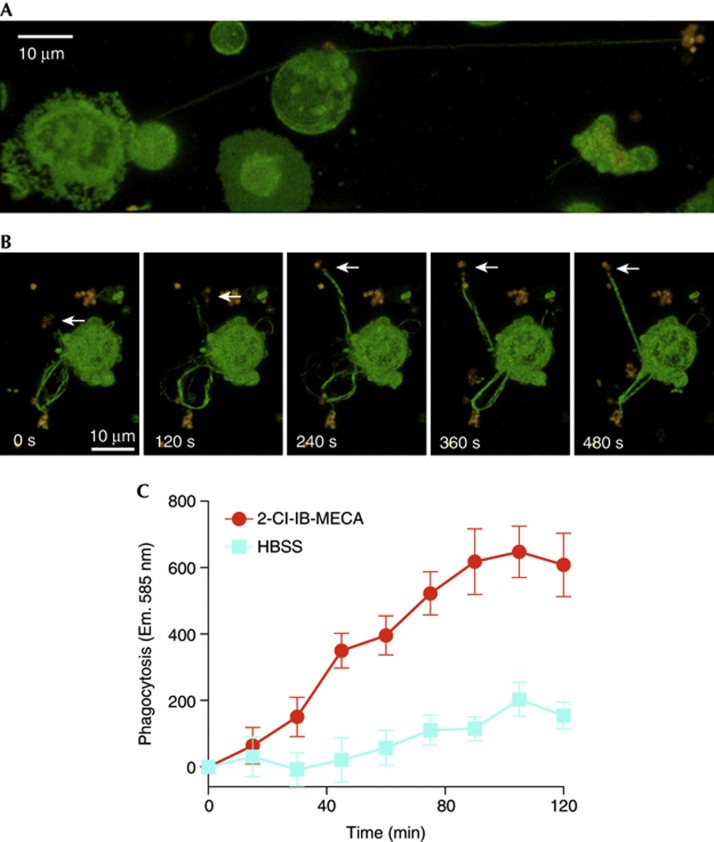
Neutrophil nanotubes enable the targeted, long-distance sampling/capture of pathogens. (**A**) Confocal imaging of Vybrant DiO-stained neutrophils that were exposed to SYTO 83-stained H. pylori revealed that cytonemes enable neutrophils to sample/capture bacteria up to 100 μm away from the cell. In addition, time-lapse imaging revealed that neutrophils are capable of simultaneously extending several cytonemes and capturing several pathogens at a time. (**B**) The spatial adaptation of cytonemes in relation to the position of motile bacteria (for example, the H. pylori cluster highlighted with white arrows) suggests that cytonemes are capable of adapting to changing spatio-temporal signals. See also supplementary Video S3 online. (**C**) Activation of A_3_ARs with 2-Cl-IB-MECA enhanced the ability of human neutrophils to phagocytose fluorescent S. aureus bioparticles. Data shown are means±s.e.m. of three individual experiments. Y axis shows relative intensity units. A_3_AR, A3-adenosine receptor; HBSS, Hank’s Buffered Salt Solution; 2-Cl-IB-MECA, 2-chloro-N^6^-(3-iodobenzyl)adenosine-5′-N-methylcarboxamide.

To further investigate the physical properties of neutrophil cytonemes and confirm that this behaviour was not restricted to one type of bacteria, experiments were performed using SYTO 83-stained *Helicobacter pylori*. As these bacteria can adhere to fibronectin-coated substrates [19], thus slowing/halting the cytoneme retraction process, they were a useful model for investigating the physical properties of these transient structures. As shown in Fig 5A, these experiments revealed that cytonemes enable the capture/sampling of pathogens up to 100 μm away from the cell. We also found that cytonemes were capable of tethering Gram-positive bacteria, such as *Staphylococcus aureus* (supplementary Fig S2 online). Neutrophils are capable of forming several cytonemes at a time, which can be used to contact several bacteria (Fig 5B). Notably, the images shown in Fig 5B suggest that the capture of pathogens by cytonemes is an active, rather than passive, process. The images depict a neutrophil extending a cytoneme towards a moving *H. pylori* cluster (indicated by the white arrows); over the course of the time series, the orientation of the cytoneme changes several times before tethering the bacterial cluster. Soon thereafter, tension simultaneously builds in all of the cytonemes that have been extended by the neutrophil, presumably as it attempts to ‘reel in’ the fibronectin-bound bacteria (supplementary Video S3 online). Previous work using epithelial cells and carboxylated beads found that the retraction of individual filipodia is dependent on receptor–ligand interactions at the filipodial tip [20]; interestingly, however, the simultaneous exertion of force suggests that all of the cytonemes extended by a single neutrophil might be controlled by a common motor/sensory complex. Finally, we tested whether A_3_ARs could be targeted to enhance the phagocytic capacity of neutrophils using pHrodo S. aureus-labelled bioparticles, finding that 2-Cl-IB-MECA stimulation enhanced the ability of neutrophils to phagocytose the bioparticles (Fig 5C).

The findings presented here not only highlight a previously unknown function of A_3_ARs, but also shed extra light on an underappreciated and poorly understood behaviour of neutrophils (that is, cytoneme-mediated capture of bacteria) [11, 12]. It has become increasingly apparent, for example, with the discovery of DNA-based traps [21], that neutrophils can deploy different strategies for the sampling, capture and clearance of invading pathogens. Our results provide further evidence that cytonemes could enable the targeted phagocytosis of pathogens that might otherwise be difficult to access. It is worth noting that recent studies of nanotubes, actin-based membrane protrusions that enable the long-range transport of signals and organelles between cells, have found that Connexin 43 hemichannels are localized at the base of these protrusions, much like the case of A_3_ARs presented here [22]. Connexin hemichannels have been shown to facilitate the release of ATP from many cell types [23], and in epithelial cells, ATP released from Connexin 26 hemichannels have been demonstrated to facilitate bacterial invasion and spreading [24]. In a related study, Romero *et al* [18] recently showed that extracellular ATP stimulates bacterial capture by epithelial cells via NMEs. In the case of neutrophils, Connexin 43 hemichannels have been shown to facilitate the release of ATP [25], which is rapidly converted to adenosine by neutrophil ecto-ATPases [7], suggesting the possible existence of an autocrine signalling pathway for the formation of cytonemes under conditions requiring a heightened surveillance state.

Our findings raise more questions for further study; for example, it would be particularly important to investigate whether different pathogens show a range of potencies for inducing cytonemes or possess mechanisms by which to avoid capture by these structures. However, taken alone, the identification of the A_3_AR as a mediator of both neutrophil chemotaxis and of cytoneme formation and function makes the receptor a potentially important therapeutic target by which to modulate neutrophil function in immunological and inflammatory disorders or infectious diseases.

## Methods

**Materials.** MRS1334 and 2-Cl-IB-MECA were obtained from Tocris Biosciences. CA200645 was obtained from CellAura Technologies (Nottingham, UK). Vybrant DiO and SYTO 83 were obtained from Life Technologies (Carlsbad, CA). Cytochalasin B, fMLP and HBSS were obtained from Sigma (St. Louis, MO). E. coli (XL1-Blue) were obtained from Agilent Technologies (Santa Clara, CA), and H. pylori (J99)/S. aureus were provided by Dr Jafar Mahdavi.

**Neutrophil isolation.** Human venous blood was collected from healthy volunteers and placed into tubes with EDTA. Neutrophils were isolated using Polymorphprep (Axis-Shield, Dundee, Scotland) according to the manufacturer’s protocol. Blood was drawn according to a protocol approved by the local ethics committee.

**Preparation of fibronectin-coated slides.** Sterile, glass-bottomed culture dishes (MatTek, Ashland, MA) and chambered coverglass slides (Nalge Nunc International, Rochester, NY) were coated with fibronectin (10 μg/ml in HBSS, Sigma). Slides/dishes were incubated at room temperature (RT) for 30 min and washed three times with HBSS before use.

**Microscope-based migration assays.** Neutrophils (2.5 × 10^6^ cells/ml) were added to fibronectin-coated 35-mm glass-bottomed dishes and incubated for 10 min at 37 °C to allow cells to settle. The dishes were then placed into a temperature-controlled stage (maintained at 37 °C) on an Axiovert S100 microscope (Carl Zeiss GmbH, Jena, Germany). Sterile Femtotips were loaded with 100 nM fMLP and connected to a FemtoJet system (Eppendorf, Hamburg, Germany). Cells were visualized using a 40 × /0.60 NA objective, and an Eppendorf Micromanipulator 5,171 was used to position the Femtotips. Continuous fMLP release was achieved by setting the compensation pressure to 100 hPa; and time-lapse images were captured using Axiovision software (v4.8; Carl Zeiss GmbH). Migration paths were traced using ImageJ (v1.44p; NIH, Bethesda, MD) with Manual Tracking (F. Cordeli, France).

**Flow cytometry.** Neutrophils were incubated with and without MRS1334 for 20 min at RT; after an additional 20 min at RT with 30 nM CA200645, cells were analysed using a Coulter FC500 flow cytometer (Beckman Coulter, Brea, CA). Median CA200645 fluorescence intensity was determined Using WinMDI software (v2.8; J. Trotter, Scripps Research Institute, La Jolla, CA). A competitive binding curve was generated using Prism (v.4.0; GraphPad Software, San Diego, CA).

**Confocal imaging.** All images shown were captured using a Zeiss LSM710 microscope (Carl Zeiss GmbH) with a × 63 plan-Apochromat/1.40NA oil DIC objective. Lasers (488 nm argon, 561 nm DPSS and 633 nm HeNe) were used to excite Vybrant DiO, SYTO 83 and CA200645. Neutrophils were labelled with Vybrant DiO according to the manufacturer’s protocol. Roughly 1 × 10^6^ labelled neutrophils were suspended in HBSS and added to a fibronectin-coated, chambered coverglass slide, which was maintained at 37 °C. Zen 2010 software (v6.0; Carl Zeiss GmbH) was used to capture image stacks, with image slices captured at 0.5 μm intervals. The image stacks were rendered as maximum intensity projections or three-dimensional images for analysis/presentation. For experiments that required the visualization of A_3_ARs, an additional 20-min incubation at 37 °C with CA200645 was performed before DiO staining. Bacteria were stained with SYTO 83 according to the manufacturer’s protocol.

**HL60 differentiation and transfection.** HL60 cells were differentiated with 1 μM all-*trans* retinoid acid (Sigma); after 108 h, the cells were transfected with the LifeAct construct (Ibidi GmbH, Munich, Germany) using a Nucleofector system (Lonza, Basel, Switzerland). After 12 h, the HL60 cells were labelled with Vybrant DiO and CA200645, and actin distribution was observed.

**Cytoneme quantification assays.** Neutrophils were pre-incubated in HBSS alone or with MRS1334 (1 μM final) or cytochalasin B (300 nM final) for 20 min or 1 h, respectively. HBSS, 2-Cl-IB-MECA (1 μM final) or *E. coli* (2.5 × 10^7^ cells/ml) were added 2.5 min after image capture was initiated. To avoid subjective bias, data were randomized and analysed by a researcher not involved in performing the experiments. Contrast and gain were adjusted to aid in visualization of the cytonemes, and the number per cell immediately before and 7.5 min after the described treatments were counted. Cytonemes were only included if they originated from a viable cell, were <1 μm in diameter and more than 5 μm long, and exhibited some movement (on the basis of time series analysis). Cells were only included if 50% or more of the cell body was visible. Structures that originated at the cell base (that is, on the coverslip) were excluded. Three experiments were excluded owing to red blood cell contamination (which made it difficult to determine cytoneme numbers and potentially altered the signalling environment). One statistical outlier that fell more than two s.d.’s outside the mean was removed.

**Phagocytosis assay.** Human neutrophils were combined in an uncoated 96-well plate with pHrodo Red S. aureus Bioparticles (Life Technologies) as specified by the manufacturer. Plates were incubated at 37 °C, and phagocytosis was assessed by measuring fluorescence intensity at 15 min intervals using a SpectraMax plate reader (Molecular Devices) at excitation and emission wavelengths of 560 and 585 nm, respectively.

**Statistical analysis.** All of the statistical analyses described in the figure legends were performed using Prism v4.0.

Supplementary information is available at EMBO *reports* online (http://www.emboreports.org).

## Supplementary Material

Supplementary Information

Supplementary Video S1

Supplementary Video S2

Supplementary Video S3

Review Process File

## References

[b1] Junger WG (2011) Immune cell regulation by autocrine purinergic signalling. Nat Rev Immunol 11: 201–212 21331080 10.1038/nri2938 PMC4209705

[b2] Elliott MR (2009) Nucleotides released by apoptotic cells act as a find-me signal to promote phagocytic clearance. Nature 461: 282–286 19741708 10.1038/nature08296 PMC2851546

[b3] Chen Y, Corriden R, Inoue Y, Yip L, Hashiguchi N, Zinkernagel A, Nizet V, Insel PA, Junger WG (2006) ATP release guides neutrophil chemotaxis via P2Y2 and A3 receptors. Science 314: 1792–1795 17170310 10.1126/science.1132559

[b4] Kronlage M (2010) Autocrine purinergic receptor signaling is essential for macrophage chemotaxis. Sci Signal 3: ra55 20664064 10.1126/scisignal.2000588

[b5] McDonald B, Pittman K, Menezes GB, Hirota SA, Slaba I, Waterhouse CCM, Beck PL, Muruve DA, Kubes P (2010) Intravascular danger signals guide neutrophils to sites of sterile inflammation. Science 330: 362–366 20947763 10.1126/science.1195491

[b6] Corriden R, Insel PA (2010) Basal release of ATP: an autocrine-paracrine mechanism for cell regulation. Sci Signal 3: re1 20068232 10.1126/scisignal.3104re1 PMC3085344

[b7] Corriden R, Chen Y, Inoue Y, Beldi G, Robson SC, Insel PA, Junger WG (2008) Ecto-nucleoside triphosphate diphosphohydrolase 1 (E-NTPDase1/CD39) regulates neutrophil chemotaxis by hydrolyzing released ATP to adenosine. J Biol Chem 283: 28480–28486 18713747 10.1074/jbc.M800039200 PMC2568933

[b8] Inoue Y, Chen Y, Hirsh MI, Yip L, Junger WG (2008) A3 and P2Y2 receptors control the recruitment of neutrophils to the lungs in a mouse model of sepsis. Shock 30: 173–177 18091570 10.1097/shk.0b013e318160dad4 PMC4212521

[b9] van der Hoeven D, Wan TC, Auchampach JA (2008) Activation of the A3 adenosine receptor suppresses superoxide production and chemotaxis of mouse bone marrow neutrophils. Mol Pharmacol 74: 685–696 18583455 10.1124/mol.108.048066 PMC2574951

[b10] Stoddart Leigh A, Vernall Andrea J, Denman Jessica L, Briddon Stephen J, Kellam B, Hill Stephen J (2012) Fragment screening at adenosine-A3 receptors in living cells using a fluorescence-based binding assay. Chem Biol 19: 1105–1115 22999879 10.1016/j.chembiol.2012.07.014 PMC3456874

[b11] Galkina SI, Molotkovsky JG, Ullrich V, Sud'ina GF (2005) Scanning electron microscopy study of neutrophil membrane tubulovesicular extensions (cytonemes) and their role in anchoring, aggregation and phagocytosis. The effect of nitric oxide. Exp Cell Res 304: 620–629 15748905 10.1016/j.yexcr.2004.12.005

[b12] Galkina SI, Romanova JM, Stadnichuk VI, Molotkovsky JG, Sud'ina GF, Klein T (2009) Nitric oxide-induced membrane tubulovesicular extensions (cytonemes) of human neutrophils catch and hold Salmonella enterica serovar Typhimurium at a distance from the cell surface. FEMS Immunol Med Microbiol 56: 162–171 19453754 10.1111/j.1574-695X.2009.00560.x

[b13] Pospieszalska MK, Ley K (2009) Dynamics of microvillus extension and tether formation in rolling leukocytes. Cell Mol Bioeng 2: 207–217 20046963 10.1007/s12195-009-0063-9 PMC2747329

[b14] Sundd P, Gutierrez E, Koltsova EK, Kuwano Y, Fukuda S, Pospieszalska MK, Groisman A, Ley K (2012) ‘Slings' enable neutrophil rolling at high shear. Nature 488: 399–403 22763437 10.1038/nature11248 PMC3433404

[b15] Jacobson KA (1998) Adenosine A3 receptors: novel ligands and paradoxical effects. Trends Pharmacol Sci 19: 184–191 9652191 10.1016/s0165-6147(98)01203-6 PMC3158240

[b16] Baraldi PG, Borea PA (2000) New potent and selective human adenosine A3 receptor antagonists. Trends Pharmacol Sci 21: 456–459 11121831 10.1016/s0165-6147(00)01581-9

[b17] Vonna L, Wiedemann A, Aepfelbacher M, Sackmann E (2007) Micromechanics of filopodia mediated capture of pathogens by macrophages. Eur Biophys J 36: 145–151 17160406 10.1007/s00249-006-0118-y

[b18] Romero S, Grompone G, Carayol N, Mounier J, Guadagnini S, Prevost M-C, Sansonetti PJ, Van Nhieu GT (2011) ATP-mediated Erk1/2 activation stimulates bacterial capture by filopodia, which precedes shigella invasion of epithelial cells. Cell Host Microbe 9: 508–519 21669399 10.1016/j.chom.2011.05.005 PMC4425278

[b19] Walz A, Odenbreit S, Mahdavi J, Boren T, Ruhl S (2005) Identification and characterization of binding properties of *Helicobacter pylori* by glycoconjugate arrays. Glycobiology 15: 700–708 15716466 10.1093/glycob/cwi049

[b20] Romero S, Quatela A, Bornschlögl T, Guadagnini S, Bassereau P, Tran Van Nhieu G (2012) Filopodium retraction is controlled by adhesion to its tip. J Cell Sci 125: 4999–5004 22899718 10.1242/jcs.104778 PMC3533388

[b21] Brinkmann V, Reichard U, Goosmann C, Fauler B, Uhlemann Y, Weiss DS, Weinrauch Y, Zychlinsky A (2004) Neutrophil extracellular traps kill bacteria. Science 303: 1532–1535 15001782 10.1126/science.1092385

[b22] Wang X, Veruki ML, Bukoreshtliev NV, Hartveit E, Gerdes H-H (2010) Animal cells connected by nanotubes can be electrically coupled through interposed gap-junction channels. Proc Natl Acad Sci USA 107: 17194–17199 20855598 10.1073/pnas.1006785107 PMC2951457

[b23] Lazarowski E (2012) Vesicular and conductive mechanisms of nucleotide release. Purinergic Signal 8: 359–373 22528679 10.1007/s11302-012-9304-9 PMC3360093

[b24] Van Nhieu GT, Clair C, Bruzzone R, Mesnil M, Sansonetti P, Combettes L (2003) Connexin-dependent inter-cellular communication increases invasion and dissemination of Shigella in epithelial cells. Nat Cell Biol 5: 720–726 12844145 10.1038/ncb1021

[b25] Eltzschig HK, Eckle T, Mager A, Küper N, Karcher C, Weissmüller T, Boengler K, Schulz R, Robson SC, Colgan SP (2006) ATP release from activated neutrophils occurs via connexin 43 and modulates adenosine-dependent endothelial cell function. Circ Res 99: 1100–1108 17038639 10.1161/01.RES.0000250174.31269.70

