# Supplementary material for: Adenosine-A3 receptors in neutrophil microdomains promote the formation of bacteria-tethering cytonemes
Source: EMBO Rep. 2013 Jul 2;14(8):726–32. doi: 10.1038/embor.2013.89 (PMC3736131; doi:10.1038/embor.2013.89)

Online Supplementary Material: Brief legends for the three supplemental video files (each in .mov format) and two supplementary figures are provided below.

Supplementary Video Legends

Supplementary Video 1: 3D reconstruction of a Vybrant DiO-labeled human neutrophil (green) using a tether to capture and retract a SYTO 83-labeled *E. coli* (orange).

Supplementary Video 2: 3D reconstruction of Vybrant DiO-labeled human neutrophils (green) exposed to SYTO 83-labeled *E. coli* (orange). Cells on the right side of the frame can be seen extending tethers towards the bacteria, while cells on the left side of the frame capture the bacteria via classical chemotaxis/phagocytosis.

Supplementary Video 3: 3D reconstruction of Vybrant DiO-labeled human neutrophils (green) exposed to SYTO 83-labeled *H. pylori* (red). The cell shown has extended several tethers to capture multiple bacteria that have adhered to the fibronectin-coated coverslip. During the course of the video, the cell extends a new tether that changes orientation several times to capture a free-floating cluster of bacteria. Once the cell has captured the bacteria, tension simultaneously builds in all of the tethers.

Supplementary Figure Legends

Supplementary Figure 1: Neutrophil cytonemes are not associated with neutrophil extracellular traps (NETs). Vybrant DiO (green)-labeled human neutrophils were imaged in the presence of the DNA stain SYTO 83 (blue). Although staining of the nuclei was observed, no DNA could be observed outside the cell in the proximity of the cytoneme, indicating that these structures are independent of DNA-based NETs.

Supplementary Figure 2: Neutrophil cytonemes are capable of tethering Gram-positive bacteria. 3D reconstruction of a Vybrant DiO-labeled human neutrophil (green) exposed to SYTO 83-labeled *S. aureus* (orange). Bacteria can be observed at the tips of two neutrophil cytonemes.

Supplemental Figure 1

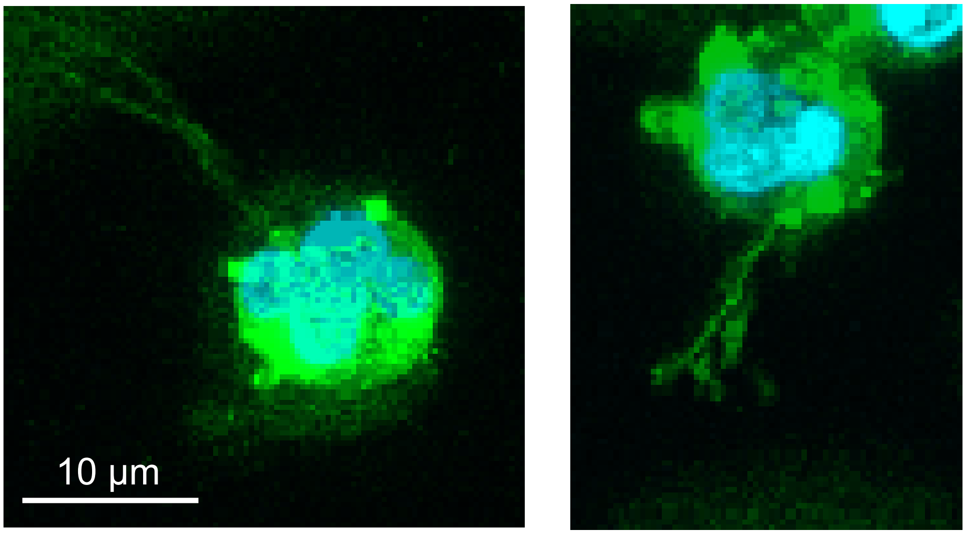

Supplemental Figure 2

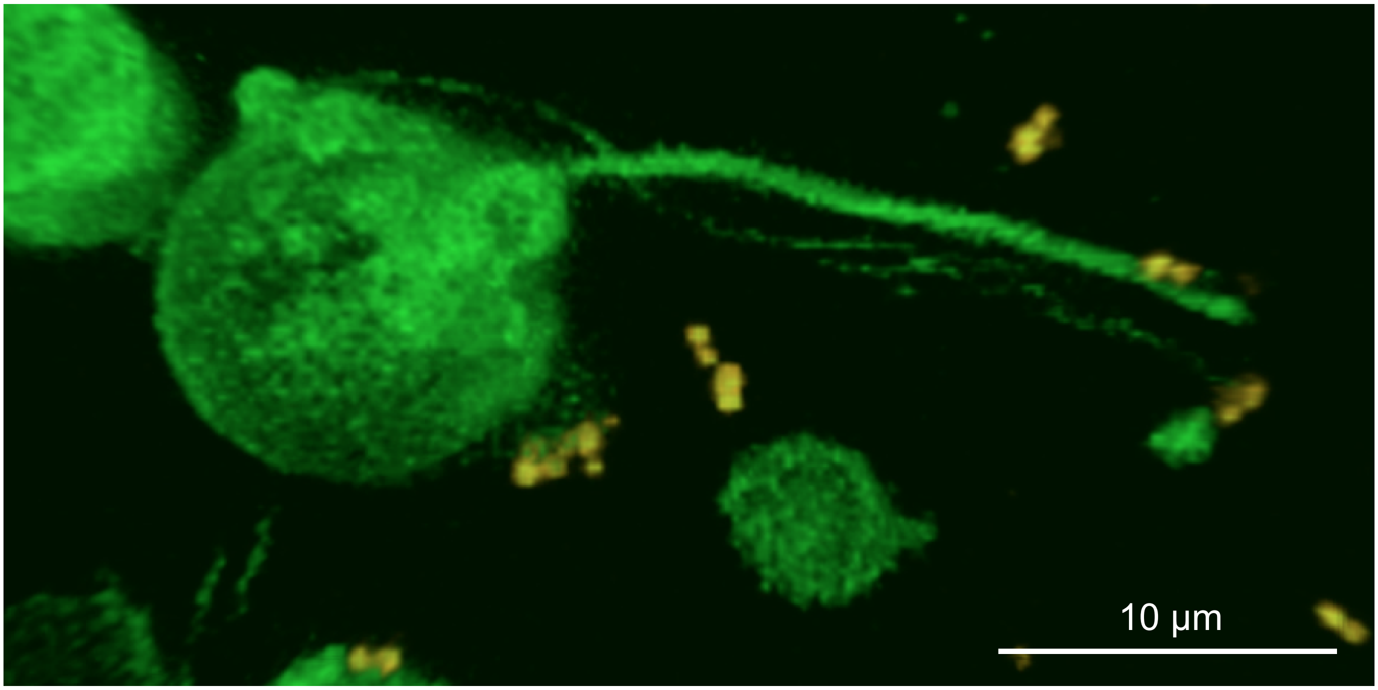

Supplement: Supplementary Information [file embor201389s1.pdf]
